# Risk of Closure Among Independent and Multihospital-Affiliated Rural Hospitals

**DOI:** 10.1001/jamahealthforum.2022.1835

**Published:** 2022-07-01

**Authors:** H. Joanna Jiang, Kathryn R. Fingar, Lan Liang, Rachel Mosher Henke

**Affiliations:** 1 Agency for Healthcare Research and Quality, Rockville, Maryland; 2 IBM, Sacramento, California; 3 IBM, Cambridge, Massachusetts

## Abstract

**Importance:**

The increase in rural hospital closures has strained access to inpatient care in rural communities. It is important to understand the association between hospital system affiliation and access to care in these communities to inform policy on this issue.

**Objective:**

To examine the association between affiliation and rural hospital closure.

**Design, Setting, and Participants:**

This cohort study used survival models with a time-dependent variable for affiliation vs independent status to assess risk of closure among a national cohort of US rural hospitals from January 2007 through December 2019. Data analysis was conducted from March to October 2021. Hospital affiliations were identified from the American Hospital Association Annual Survey and Irving Levin Associates and closures from the University of North Carolina Sheps Center (Chapel Hill). Additional covariates came from the Healthcare Cost and Utilization Project State Inpatient Databases and other national sources.

**Exposures:**

Affiliation with another hospital or multihospital health system.

**Main Outcomes and Measures:**

Closure was the main outcome. The models included hospital, market, and utilization characteristics and were stratified by financial distress in 2007.

**Results:**

Among 2237 rural hospitals operating in 2007, 140 (6.3%) had closed by 2019. The proportion of rural hospitals that were independent decreased from 68.9% in 2007 to 47.0% in 2019; the proportion that were affiliated increased from 31.1% to 46.7%. Among financially distressed hospitals in 2007, affiliation was associated with lower risk of closure compared with being independent (adjusted hazard ratio [aHR], 0.49; 95% CI, 0.26-0.92). Conversely, among hospitals that were financially stable in 2007, affiliation was associated with higher risk of closure compared with being independent (aHR, 2.36; 95% CI, 1.20-4.62). For-profit ownership was also strongly associated with closure for hospitals that were financially stable in 2007 (aHR, 4.08; 95% CI, 1.86-8.97).

**Conclusions and Relevance:**

The results of this cohort study suggest that affiliations may be associated with lower risk of closure for some rural hospitals in financial distress. However, among initially financially stable hospitals, an increased risk of closure for hospitals associated with affiliation and proprietary ownership raises concerns about the association of affiliation with closures in some circumstances. Policy interventions to stabilize inpatient care in rural areas should account for these findings.

## Key Points

**Question:**

Is affiliation with another hospital or multihospital system associated with lower risk of closure in rural areas?

**Findings:**

In this 13-year cohort study from 2007 to 2019 among rural US hospitals that faced financial distress in 2007, affiliation was associated with a lower risk of closure compared with being independent. Conversely, among hospitals with financial stability in 2007, affiliation was associated with a higher risk of closure compared with being independent.

**Meaning:**

The study results suggest that affiliation may protect some financially distressed rural hospitals from closure, but the higher risk of closure among hospitals that were initially financially stable potentially raises concerns about the consequences of affiliation for some types of hospitals.

## Teaser

This cohort study examines thhe association between affiliation and rural hospital closure.

## Introduction

Health care in rural areas is at a crossroads. Rural communities have greater health needs than urban areas but fewer health care resources.^1,2,3^ Rural hospitals have struggled with attracting and retaining clinicians, who generally prefer metropolitan areas.^4^ They also face high fixed costs to provide services and adopt technology, primarily because of low patient volumes to absorb these costs.^5^ Further exacerbating the issue of insufficient volume, individuals residing in rural areas may bypass their local hospital for higher technology hospitals further away.^6^ Patients in rural areas are also more likely to be uninsured or covered by public insurance programs.^7^ Because of these factors, rural hospitals have faced more financial distress than urban hospitals as the gap in profitability between these 2 hospital types has widened.^8^ During the past 10 years, more than 100 rural hospitals in the US have closed.^5,9^

The number of affiliations between rural hospitals and other hospitals or multihospital systems has also increased during the past decade.^5,10^ Affiliations may be a viable alternative to closure for hospitals that are experiencing financial distress.^11^ However, it is unclear whether hospitals that become affiliated are protected from closure. Prior research found that rural hospitals with lower levels of profitability and less ability to cover debt payments were more likely to merge and be acquired.^12^ However, mergers may not necessarily improve profitability, capital structure, and debt payments.^11^ Further, mergers appear to be associated with a reduction of service lines, including maternity and surgical care.^13,14,15^ In some cases, there have been reports that hospital closure was a business decision at the system level that did not prioritize community needs.^16^

However, affiliation with another hospital or multihospital system may shelter hospitals from closure. Compared with independent hospitals, affiliated hospitals may have more ability to tap into resources that are available from the system.^17^ These hospitals may have better access to shared staff and other administrative resources to lower fixed costs.^18,19^ They may also have better access to specialized technology and more advanced information management capacity to help rural hospitals improve operating efficiency.^20^ Thus, greater access to resources for affiliated hospitals may protect them from closure.

The primary objective of this cohort study was to estimate risk of closure associated with hospital affiliation among rural US hospitals. First, we used data from all 50 states to examine how the composition of rural hospitals changed from January 2007 to December 2019 in terms of remaining independent, becoming affiliated, or closing. Second, we drew from multiple sources to describe hospitals that were already affiliated at baseline in 2007, those that were originally independent but became affiliated during the study period, and those that were independent until closure or 2019. Third, we estimated survival models to assess the risk of closure that was associated with affiliated vs independent status.

Financially distressed rural hospitals may have unique factors associated with survival because of their need to expand sources of income to cover debt. Thus, we stratified the survival models by financial status. Because new affiliations may differ from more mature affiliations,^19^ we also conducted an analysis that was limited to the cohort of hospitals that were independent in 2007 to examine whether the association between new affiliations and closure was consistent with findings from the full sample. The results of this cohort study potentially inform policy makers who are determining how to address the current health care crisis in rural areas.

## Methods

### Data Sources and Study Population

We identified community general acute care hospitals in rural areas of the US that were open in 2007 from the American Hospital Association (AHA) Annual Survey, excluding those that closed in 2007. In line with the AHA, community hospitals exclude federal hospitals and hospitals inaccessible to the public. *Rural* was defined as being in a zip code that was eligible for funding from the Federal Office of Rural Health Policy.^21^ We identified hospital mergers and acquisitions between 2007 and 2019 from Irving Levin Associates, augmenting this information with data on self-reported affiliation from the AHA.^22^ Hospital closures from 2007 through 2019 were obtained from the University of North Carolina Sheps Center (Chapel Hill).^23^

Additional hospital and market characteristics came from several sources. Inpatient utilization for rural hospitals was obtained from the Healthcare Cost and Utilization Project (HCUP) State Inpatient Databases (SID) from 2007 to 2019 for 39 states.^24^ These data were linked to hospital financial data from the 2007 to 2019 US Centers for Medicare & Medicaid Services cost reports.^25^ Community measures corresponding to the hospital’s market came from the 2007 to 2019 American Community Survey files.^26^ The number of nearby hospitals and the Herfindahl-Hirschman Index, a measure of competition, came from the HCUP hospital market structure files.^27^ Medicaid expansion status data came from the Kaiser Family Foundation.^28^

The Agency for Healthcare Research and Quality human protections administrator determined this project did not constitute research involving human participants; thus, it was not required by the agency to be submitted to an institutional review board. This study follows Strengthening the Reporting of Observational studies in Epidemiology (STROBE) reporting guidelines.

### Primary Dependent and Independent Variables

Using data from Irving Levin Associates, the AHA, the Sheps Center, and internet searches, when inconsistencies across sources were found, we classified hospitals in each year as *independent*, *affiliated*, or *closed*. *Affiliated* was a broad group that included mergers and acquisitions documented by Irving Levin Associates, as well as self-reported system membership according to the AHA, which may have also included formal mergers and acquisitions as well as informal affiliations that did not involve change in governance.^29^ The AHA membership definition included managed service contracts. We identified these arrangements using the system name provided in the survey and classified these hospitals as independent because a hospital remained independently owned according to these contracts. Details are provided in eTable 1 in the Supplement, including a breakout of hospitals with managed service contracts. Closure was the primary dependent variable.

### Other Covariates

Financial distress was measured using the Altman *z* score, which has been used during the past 4 decades in multiple industries, including the health care sector.^30^ There have been updates to the definition as it applies to entities of different ownership (ie, private, for profit, and nonprofit).^31^ We used the version (ie, *z* double prime) used by McCay et al^32^ because it was developed for industries that include nonprofit entities. The composite score was calculated by adding 4 components measuring liquidity, profitability, efficiency, and productivity. We used cut points defined by prior literature^32^ to categorize hospitals as financially distressed or concerned (≤2.8, hereafter referred to as *distressed*) and stable (>2.8).^32^ Other hospital characteristics, measured in 2007, included number of beds, critical access status, ownership, and region. We also obtained state Medicaid expansion status data. Market characteristics included mean household income, percentage of the population with health insurance, percentage of the population that was self-identified as White, unemployment rate, mean resident age, market share of inpatient stays, the number of hospitals within a 15-mile fixed radius, and the Herfindahl-Hirschman Index.

Utilization characteristics included discharge volume, length of stay, mean cost per stay, distance to the hospital, payer mix, and service line mix from the SID and annual occupancy rate from the AHA. Hospital charges were converted to costs using the HCUP cost-to-charge ratios.^33^ Payer mix is based on the primary expected payer.^34^ Service line mix, which characterizes the reason for the stay, is based on mutually exclusive hierarchical groupings (ie, maternal/neonatal, mental and/or substance use disorder [MSUD], injury, surgical, and general medical) that are defined according to HCUP specifications.^35,36^

### Statistical Analysis

We first assessed changes in the composition of rural hospitals (independent, affiliated, and closed) during each year from 2007 to 2019. Hospital composition over time was presented for all US rural hospitals in the AHA, regardless of whether the hospital was in the HCUP SID. However, all subsequent tables and figures are based on the subset of hospitals with utilization statistics from the SID for 39 states.

Second, we compared select characteristics of hospitals at baseline, by whether the hospital was already affiliated in 2007, if it was independent in 2007 but became affiliated during the study period, or if it was independent in 2007 until closure or 2019. These groups were compared using 2-sided χ^2^ and *t* tests and a level of significance of .05.

Third, we examined changes in utilization characteristics from 2007 until closure or 2019 by whether the hospital was already affiliated in 2007, independent in 2007 but became affiliated during the study period, or independent until closure or 2019. Because closing hospitals contributed fewer years to the study, we calculated the annual average percentage change (AAPC) between 2007 and 2019 for each hospital that did not close or between 2007 and the last year that data were available for each hospital that did close. For hospitals that became affiliated, we also calculated the AAPC from 2007 to the year of affiliation and from the year of affiliation to the year of closure or 2019. Comparisons across groups were made using 2-sided χ^2^ and *t* tests. The descriptive results assessed significance at the levels of .01, .05, and .10.

Finally, we estimated hazard ratios (HRs) and 95% CIs using Cox survival models that assessed the risk of closure associated with affiliation status as adjusted for baseline hospital, market, and utilization characteristics. We ran models overall and by financial distress in 2007, including all hospitals operating in 2007 (ie, those that were already affiliated and those that were independent in 2007). We also conducted a subanalysis of hospitals that were independent in 2007 (including those that did and did not become affiliated during the study period). Because affiliation status changed over time, we treated it as a time-dependent variable (eg, a hospital contributed time in the independent group before becoming affiliated). The models adjusted for hospital, market, and utilization characteristics that were measured in 2007. Proportional HRs were presented. The results of tests for the proportional hazard assumption are provided in eTable 2 in the Supplement. Statistical analyses were conducted using SAS, version 9.4 (SAS Institute).

## Results

A total of 2237 rural US hospitals operating in 2007 according to AHA data were included in this study. A subset of 1772 hospitals linked to HCUP SID was included in all analyses.

### Changing Composition of Rural Hospitals From 2007 to 2019

**Figure 1.**
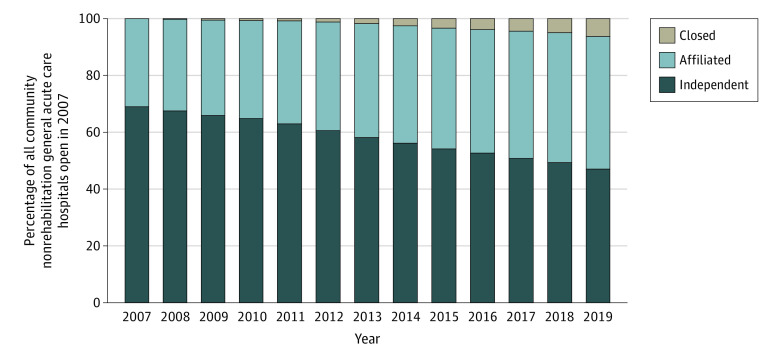
Trends in the Composition of US Rural Hospitals Open in 2007 The data include rural community general acute care hospitals open in the US throughout 2007 from the American Hospital Association (AHA) Annual Survey, excluding those that closed in 2007.

In 2007, 1542 rural hospitals (68.9%) were independent (Figure 1). Between 2007 and 2019, the percentage of independent hospitals steadily declined to 47.0% as affiliations and closures increased. By 2019, of the 2237 hospitals that were originally open in 2007, 1046 (46.7%) were affiliated and 140 (6.3%) had closed.

### Hospital Characteristics by Affiliation Status

Table 1 presents hospital organizational and utilization characteristics as measured in 2007. At baseline, independent hospitals were more likely to be publicly owned (442 [54.6%]), whereas both groups of affiliated hospitals were more likely to be private nonprofit (68.3% and 59.7% for already and newly affiliated, respectively). About two-thirds of independent hospitals were critical access hospitals (496 [61.2%]) compared with about half of rural hospitals that were already or became affiliated (44.9% and 46.5%, respectively). Affiliated hospitals had more beds on average and were more likely than independent hospitals to be in the South. The West had a higher proportion of independent hospitals. Hospitals that were already affiliated were more likely to offer maternal and neonatal care than independent hospitals (71.7% vs 61.1%). Both already and newly affiliated hospitals were more likely to offer MSUD and surgical care compared with independent hospitals (>80% vs 74%).

**Table 1.** Comparisons of Hospitals by Affiliation Status and Baseline Characteristics in 2007^a^

Characteristic	No. (%)^b^			
	All rural hospitals in HCUP, 2007	Already affiliated in 2007	Became affiliated during study period	Independent until closure or 2019
Total, No.	1772 (100)	575 (100)	387 (100)	810 (100)
Financially distressed	443 (25.0)	165 (28.7)^c^	105 (27.1)^c^	173 (21.4)
Ownership				
Public	634 (35.8)	72 (12.5)^c^	120 (31.0)^c^	442 (54.6)
Private nonprofit	964 (54.4)	393 (68.3)^c^	231 (59.7)^c^	340 (42.0)
Private for profit	174 (9.8)	110 (19.1)^c^	36 (9.3)^c^	28 (3.5)
Critical access hospital	934 (52.7)	258 (44.9)^c^	180 (46.5)^c^	496 (61.2)
No. of beds, mean (SD)	55.7 (56.4)	63.3 (69.2)^c^	63.9 (54.2)^c^	46.4 (44.8)
Region				
Northeast	106 (6.0)	22 (3.8)	38 (9.8)^c^	46 (5.7)
Midwest	795 (44.9)	244 (42.4)	175 (45.2)	376 (46.4)
South	605 (34.1)	215 (37.4)^c^	162 (41.9)^c^	228 (28.1)
West	266 (15.0)	94 (16.3)	12 (3.1)^c^	160 (19.8)
Service line provision, %^d^				
Maternal/neonatal	1164 (65.7)	412 (71.7)^c^	257 (66.4)	495 (61.1)
MSUD	1395 (78.7)	473 (82.3)^c^	322 (83.2)^c^	600 (74.1)
Injury	1653 (93.3)	541 (94.1)^c^	373 (96.4)^c^	739 (91.2)
Surgical	1419 (80.1)	496 (86.3)^c^	326 (84.2)^c^	597 (73.7)
General medical	1771 (99.9)	574 (99.8)	387 (100)	810 (100)

Abbreviations: HCUP, Healthcare Cost and Utilization Project; IP, inpatient; MSUD, mental and/or substance use disorder.

^a^ Data from the Agency for Healthcare Research and Quality, HCUP, state IP databases, and hospital market structure files; as well as the American Community Survey; American Hospital Association Annual Survey; US Centers for Medicare & Medicaid Services, cost reports; Irving Levin Associates, Inc, Mergers and Acquisitions Database; Kaiser Family Foundation; and University of North Carolina Sheps Center.

^b^ Percentages may not add to 100% because of rounding or because of missing data.

^c^ *P* value <.05 from χ^2^ or *t* test comparing affiliated with independent hospitals.

^d^ Defined as having 5 or more stays in the service line annually.

Finally, a greater proportion of hospitals that became affiliated or were already affiliated in 2007 were experiencing financial distress compared with independent hospitals (27.1% and 28.7% vs 21.4%). However, the percentage of hospitals in financial distress increased from 25.0% in 2007 to 30.2% in 2019 overall and increased among hospitals that became affiliated (27.1% to 37.6%) and those that were independent until 2019 (21.4% to 29.3%). However, they did not increase for hospitals that were already affiliated (28.7% to 26.6%). eTable 3 in the Supplement presents the detailed statistics for 2019.

**Figure 2.**
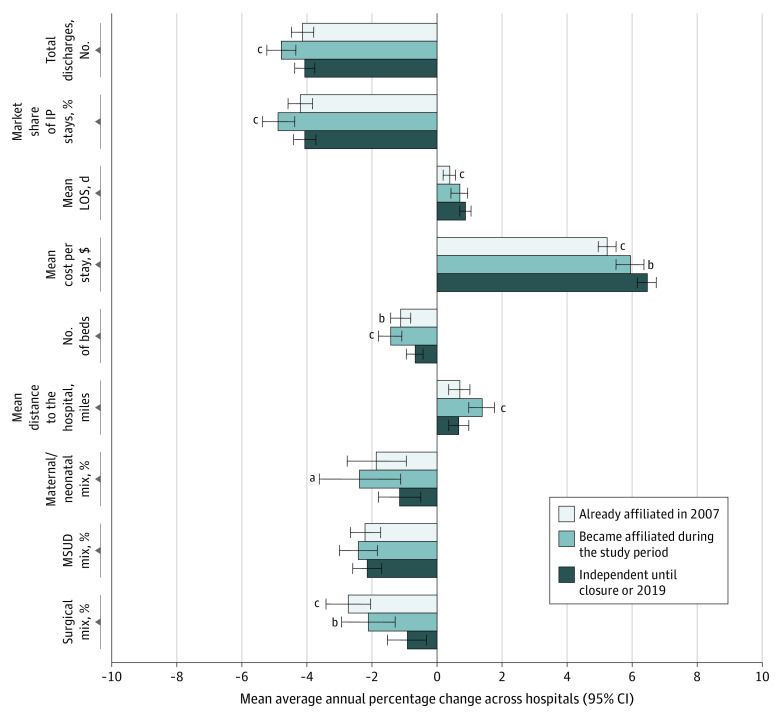
Changes in Inpatient Utilization From 2007 Until Closure or 2019 Excludes outlier hospitals where the average annual percentage change was less than the first percentile or more than the 99th percentile. Average annual percentage change in utilization characteristics from 2007 until closure or 2019 was calculated for each hospital with nonmissing values in both years and a nonzero value in 2007. IP indicates inpatient; LOS, length of stay; MSUD, mental and/or substance use disorder. ^a^*P* < .10 for difference with independent hospitals. ^b^*P* < .05 for difference with independent hospitals. ^c^*P* < .01 for difference with independent hospitals.

Figure 2 displays changes in hospital utilization from 2007 through 2019 for hospitals that remained open and from 2007 until the last year data were available for hospitals that closed. Because hospitals that closed contributed fewer years to the study, average annualized changes are shown (ie, the AAPC averaged across all hospitals). For hospitals that became affiliated, the average annual percentage decreases in total volume and market share were greater than those for independent hospitals (4.8% vs 4.1% decrease and 4.9% vs 4.1% decrease). At the same time, they had a lower increase in the mean cost per stay (5.9% vs 6.5% increase) and a greater increase in the average distance traveled for inpatient care (1.4% vs 0.7% increase). Compared with independent hospitals, those that were already affiliated also had a lower increase in average cost, as well as a lower increase in average length of stay. eFigure 1 in the Supplement displays the same statistics for hospitals that became affiliated but calculates a separate AAPC from 2007 through the year of the affiliation and from the year of the affiliation to the year of closure or 2019.

### Factors Associated With Hospital Closure

Table 2 presents adjusted HRs (aHRs) for the association between affiliation and closure overall and by financial distress in 2007. Overall, affiliation was not associated with greater or lower risk of closure compared with being independent. However, among hospitals that experienced financial distress in 2007, affiliation was associated with lower risk of closure (aHR, 0.49; 95% CI, 0.26-0.92). There was no association between new affiliation (ie, affiliation that occurred after 2007) and risk of closure when we limited the cohort of hospitals to those that were independent in 2007. Conversely, among hospitals that were financially stable in 2007, affiliation was associated with increased risk of closure (aHR, 2.36; 95% CI, 1.20-4.62), and this was consistent in the subset of hospitals that were independent in 2007 (aHR, 2.89; 95% CI, 1.07-7.83).

**Table 2.** Proportional HRs for the Association Between the Time-Dependent Variable for Affiliation Status and Closure

Cohort and model stratified by financial distress in 2007^a^	Adjusted HR (95% CI) comparing affiliated vs independent status		
	Hospitals, No.	HR (95% CI)	*P* value
Cohort of all hospitals operating in 2007			
All hospitals	1719	0.96 (0.60-1.52)	.85
Financially distressed hospitals	437	0.49 (0.26-0.92)	.03
Financially stable hospitals	1282	2.36 (1.20-4.62)	.01
Cohort of independent hospitals operating in 2007^b^			
All hospitals	1172	1.60 (0.88-2.92)	.12
Financially distressed hospitals	274	1.05 (0.48-2.31)	.91
Financially stable hospitals	898	2.89 (1.07-7.83)	.04

Abbreviation: HR, hazard ratio.

^a^ The total amount of follow-up time included 22 537 hospital-years, with an average year of closure of 2015. Models were adjusted for financial distress (model 1 only), as well as hospital characteristics (number of beds, critical access status, hospital region, ownership, whether the hospital was in a state that expanded Medicaid), market characteristics (market share; median age; mean household income; percentage of residents with health insurance, who were self-identified as White as drawn from the American Community Survey, and who were unemployed), and utilization characteristics (total discharge volume, occupancy rate, average length of stay, average cost per stay, distance traveled to the hospital, payer mix, and service line mix). Each characteristic was measured at baseline in 2007. Of the initial 1772 hospitals, 53 were excluded from the model if they had missing data on financial distress (n = 24) or other independent variables measured in 2007. Results from the full models are shown in the eTable 4 in the Supplement.

^b^ Subset that included hospitals that were independent in 2007, which later became affiliated or remained independent until 2019 or closure. Excluded hospitals that were affiliated in 2007. Thus, the association between affiliation and closure applied to hospitals that became affiliated during the study period.

Other associations with closure are presented in eTable 4 and eFigures 2 to 5 in the Supplement. Financial distress was one of the factors most strongly associated with closure (aHR, 1.98; 95% CI, 1.29-3.04). Private for-profit ownership also was a risk factor, particularly for financially stable hospitals (aHR, 4.08; 95% CI, 1.86-8.97).

## Discussion

The results of this cohort study suggest that the landscape of rural hospitals in the US has changed substantially since 2007. Approximately 6% of rural hospitals that were open in 2007 have now closed. Affiliations and financial distress have increased substantially, whereas market shares have declined. Downsizing was observed across the board in terms of decreases in number of beds and total volume of inpatient stays. During the study period, rural hospitals decreased maternal and neonatal, MSUD, and surgical inpatient services. Consistent with other research,^13^ affiliated hospitals were more likely to offer these specialized services than independent hospitals, but the average annual decrease in the percentage of surgical stays at the hospital was greater for affiliated than for independent hospitals.

Overall, the risk of closure associated with being affiliated vs being independent in any given year was similar. However, among hospitals that were financially stable in 2007, affiliation was associated with increased risk of closure compared with being independent, and this association held for new affiliations (ie, those occurring after 2007). Notably, hospitals that became affiliated during the study period had a higher rate of financial distress at the end of the study period, as well as a greater reduction in total inpatient volume and market share than independent hospitals, suggesting that these hospitals may have not performed well even after joining a system or merging with another hospital. Additionally, private for-profit ownership was associated with closure among financially stable hospitals. Thus, it is possible that, in some cases, hospital closure was a business decision that did not prioritize community needs.^16^

Conversely, among financially distressed hospitals, affiliations were associated with a lower risk of risk of closure compared with being independent. This finding did not hold in the subanalysis of hospitals that were independent in 2007, suggesting the protective association may have been associated with mature affiliations (ie, hospitals already affiliated in 2007). This finding supports the notion that some financially distressed affiliated rural hospitals may have protection from closure from their expanded access to resources. It is also consistent with research on data from the 1980s that found rural hospitals that were affiliated with certain types of systems (ie, non–investor-owned) had a higher probability of survival.^37^ Further, to avert the risk of closure, system-affiliated hospitals may adjust their operations through downsizing and improving efficiency. Hospitals with mature affiliations may have other systematic differences compared with hospitals that remain independent that were not observable and thus were not adjusted for in the study models.

### Strengths and Limitations

This study had several strengths and limitations. We examined affiliation trends by combining national merger and acquisition data with the AHA data set, which covered all states. We estimated risk of closure using all-payer utilization data from most US states. Notwithstanding, this study was descriptive in the sense that we identified hospital characteristics associated with closure but could not infer causality. There may have been unmeasurable differences between hospitals that were already affiliated, newly affiliated, and independent throughout the study period that were associated with outcomes. The study included formal and informal system memberships as affiliations. We could not examine whether findings varied by membership type because hospitals did not consistently report the nature of their system membership in AHA data. Finally, we did not examine the type of the affiliated system (ie, for profit vs nonprofit), which could also be associated with the risk of closure, as found by Halpern et al.^36^

## Conclusions

In this cohort study of US rural hospitals, multihospital system affiliation was not consistently a protective factor for closure. System affiliation was associated with a lower risk of closure for financially distressed hospitals but with a higher risk of closure for hospitals that were initially financially stable. Policy interventions designed to sustain rural hospitals should consider the different situations of affiliated and independent hospitals.

## Notes

Supplement. **eTable 1. **Organizational Status of U.S. Rural Hospitals Open in 2007 (N=2,237), Through 2019 **eTable 2. **Tests for the Proportional Hazard Assumption **eTable 3. **Comparisons of Hospitals by Affiliation Status in 2007 and 2019 **eFigure 1. **Changes in Inpatient Utilization Before and After the Affiliation for Hospitals that Became Affiliated During the Study Period **eTable 4. **Proportional Hazard Ratios Predicting Risk of Closure (Full Models) **eFigure 2. **Unadjusted Survival Curves Measuring the Percentage of Hospitals Still Operating Through 2019, by Financial Distress in 2007 **eFigure 3. **Unadjusted Survival Curves Measuring the Percentage of Hospitals Still Operating Through 2019, by Ownership Overall and by Financial Distress in 2007 **eFigure 4. **Unadjusted Survival Curves Measuring the Percentage of Hospitals Still Operating Through 2019, by Critical Access Status Overall and by Financial Distress in 2007 **eFigure 5. **Unadjusted Survival Curves Measuring the Percentage of Hospitals Still Operating Through 2019, by Region Overall and by Financial Distress in 2007
