# Supplementary material for: Risk of Closure Among Independent and Multihospital-Affiliated Rural Hospitals
Source: JAMA Health Forum. 2022 Jul 1;3(7):e221835. doi: 10.1001/jamahealthforum.2022.1835 (PMC9250050; doi:10.1001/jamahealthforum.2022.1835)
Supplement: Supplement. — eTable 1. Organizational Status of U.S. Rural Hospitals Open in 2007 (N=2,237), Through 2019 eTable 2. Tests for the Proportional Hazard Assumption eTable 3. Comparisons of Hospitals by Affiliation Status in 2007 and 2019 eFigure 1. Changes in Inpatient Utilization Before and After the Affiliation for Hospitals that Became Affiliated During the Study Period eTable 4. Proportional Hazard Ratios Predicting Risk of Closure (Full Models) eFigure 2. Unadjusted Survival Curves Measuring the Percentage of Hospitals Still Operating Through 2019, by Financial Distress in 2007 eFigure 3. Unadjusted Survival Curves Measuring the Percentage of Hospitals Still Operating Through 2019, by Ownership Overall and by Financial Distress in 2007 eFigure 4. Unadjusted Survival Curves Measuring the Percentage of Hospitals Still Operating Through 2019, by Critical Access Status Overall and by Financial Distress in 2007 eFigure 5. Unadjusted Survival Curves Measuring the Percentage of Hospitals Still Operating Through 2019, by Region Overall and by Financial Distress in 2007 [file jamahealthforum-e221835-s001.pdf]

Supplemental Online Content

Jiang HJ, Fingar KR, Liang L, Henke RM. Risk of closure among independent and multihospital-affiliated rural hospitals. *JAMA Health Forum*. 2022;3(7):e221835. doi:10.1001/jamahealthforum.2022.1835

eTable 1. Organizational Status of U.S. Rural Hospitals Open in 2007 (N=2,237), Through 2019

eTable 2. Tests for the Proportional Hazard Assumption

eTable 3. Comparisons of Hospitals by Affiliation Status in 2007 and 2019

eFigure 1. Changes in Inpatient Utilization Before and After the Affiliation for Hospitals that Became Affiliated During the Study Period

eTable 4. Proportional Hazard Ratios Predicting Risk of Closure (Full Models)

eFigure 2. Unadjusted Survival Curves Measuring the Percentage of Hospitals Still Operating Through 2019, by Financial Distress in 2007

eFigure 3. Unadjusted Survival Curves Measuring the Percentage of Hospitals Still Operating Through 2019, by Ownership Overall and by Financial Distress in 2007

eFigure 4. Unadjusted Survival Curves Measuring the Percentage of Hospitals Still Operating Through 2019, by Critical Access Status Overall and by Financial Distress in 2007

eFigure 5. Unadjusted Survival Curves Measuring the Percentage of Hospitals Still Operating Through 2019, by Region Overall and by Financial Distress in 2007

This supplemental material has been provided by the authors to give readers additional information about their work.

© 2022 Jiang HJ et al. *JAMA Health Forum.*

eTable 1. Organizational Status of U.S. Rural Hospitals Open in 2007 (N=2,237), Through 2019

Status, %	2007	2008	2009	2010	2011	2012	2013	2014	2015	2016	2017	2018	2019
Independent	59.1	57.6	56.3	55.8	54.1	52.3	51.1	49.6	47.9	46.8	45.3	43.5	42.3
Affiliated[a]	30.2	31.0	31.9	32.2	32.7	33.8	34.5	34.6	34.4	34.5	34.8	34.8	34.8
Managed[b]	9.8	9.9	9.6	9.0	8.9	8.2	7.0	6.5	6.3	5.9	5.5	5.8	4.7
Merged[c]	0.8	1.3	1.7	2.3	3.5	4.6	5.6	6.8	8.0	8.9	10.1	11.0	11.9
Closed	0.0	0.2	0.6	0.7	0.8	1.2	1.8	2.5	3.4	3.9	4.4	5.0	6.3

Notes: The affiliated, managed, and merged categories were combined into the affiliated group in the main analysis. The denominator is the same (2,237) for each column.

[a] Self-reported system affiliation with a multihospital system in the American Hospital Association Annual Survey.

[b] Self-reported system affiliation with a firm specializing in managed services contracts in the American Hospital Association Annual Survey. In the main analysis, these hospitals were grouped with independent hospitals because they are independently owned.

[c] Merger or acquisition between 2007 through 2019 documented in Irving Levin Associates, Mergers and Acquisitions Database.

Source: American Hospital Association Annual Survey, 2007-2019; Irving Levin Associates, Inc., Mergers and Acquisitions Database, 2007-2019; University of North Carolina Sheps Center, 2007-2019

© 2022 Jiang HJ et al. *JAMA Health Forum.*

eTable 2. Tests for the Proportional Hazard Assumption

Model	*P* value for the interaction between affiliation*log(time)
**Cohort of all hospitals operating in 2007**	
1a. Total	0.3831
1b. Financially distressed	0.1193
1c. Financially stable	0.2771
**Cohort of independent hospitals operating in 2007[a]**	
2a. Total	0.9175
2b. Financially distressed	0.4560
2c. Financially stable	0.8060

Note: Models were adjusted for financial distress (Model 1 only), as well as hospital characteristics (bed size, critical access status, hospital region, ownership, whether the hospital was in a state that expanded Medicaid), market characteristics (market share; median age; mean household income; percentage of residents with health insurance, who were White, and who were unemployed), and utilization characteristics (total discharge volume, occupancy rate, average length of stay, average cost per stay, distance traveled to the hospital, payer mix, and service line mix). Each characteristic was measured at baseline in 2007. Results from the full models are shown in eTable 3.

[a] Stratified model that includes hospitals that were independent in 2007 and later became affiliated and hospitals that were independent in all years. The association between affiliation and closure applies to hospitals that became affiliated during the study period.

Source: Agency for Healthcare Research and Quality, Healthcare Cost and Utilization Project, State Inpatient Databases, 2007-2019, and Hospital Market Structure Files, 2006 and 2018; American Community Survey, 2007-2019; American Hospital Association Annual Survey, 2007-2019; Centers for Medicare & Medicaid Services, Cost Reports, 2007-2019; Irving Levin Associates, Inc., Mergers and Acquisitions Database, 2007-2019; Kaiser Family Foundation; University of North Carolina Sheps Center, 2007-2019.

© 2022 Jiang HJ et al. *JAMA Health Forum.*

eTable 3. Comparisons of Hospitals by Affiliation Status in 2007 and 2019

Characteristic	2007				2019			
	All rural hospitals in HCUP, 2007	Already affiliated in 2007	Became affiliated during the study period	Independent until closure or 2019	All rural hospitals in HCUP, 2019	Already affiliated in 2007	Became affiliated during the study period	Independent until 2019
**Total, No.**	1,772 (100)	575 (100)	387 (100)	810 (100)	1,665 (100)	541 (100)	362 (100)	762 (100)
**Hospital**								
Financially distressed, No. (%)	443 (25.0)	165 (28.7) *	105 (27.1) *	173 (21.4)	503 (30.2)	144 (26.6)	136 (37.6) *	223 (29.3)
Ownership, No. (%)								
Public	634 (35.8)	72 (12.5) *	120 (31.0) *	442 (54.6)	549 (33)	66 (12.2) *	66 (18.2) *	417 (54.7)
Private nonprofit	964 (54.4)	393 (68.3) *	231 (59.7) *	340 (42.0)	962 (57.8)	368 (68.0) *	265 (73.2) *	329 (43.2)
Private for-profit	174 (9.8)	110 (19.1) *	36 (9.3) *	28 (3.5)	154 (9.2)	107 (19.8) *	31 (8.6) *	16 (2.1)
Critical access hospital, No. (%)	934 (52.7)	258 (44.9) *	180 (46.5) *	496 (61.2)	972 (58.4)	269 (49.7) *	187 (51.7) *	516 (67.7)
Bed size, mean (SD)	55.7 (56.4)	63.3 (69.2) *	63.9 (54.2) *	46.4 (44.8)	51.3 (56.9)	56.4 (61.4) *	57.7 (67.7) *	44.6 (46.4)
Region, No. (%)								
Northeast	106 (6.0)	22 (3.8)	38 (9.8) *	46 (5.7)	96 (5.8)	20 (3.7)	36 (9.9) *	40 (5.2)
Midwest	795 (44.9)	244 (42.4)	175 (45.2)	376 (46.4)	774 (46.5)	235 (43.4)	169 (46.7)	370 (48.6)
South	605 (34.1)	215 (37.4) *	162 (41.9) *	228 (28.1)	537 (32.3)	193 (35.7) *	145 (40.1) *	199 (26.1)
West	266 (15.0)	94 (16.3)	12 (3.1) *	160 (19.8)	258 (15.5)	93 (17.2)	12 (3.3) *	153 (20.1)
Medicaid expanded, No. (%)	0 (0.0)	0 (0.0)	0 (0.0)	0 (0.0)	921 (55.3)	305 (56.4)	217 (59.9) *	399 (52.4)
**Market, mean (SD)**								
Mean household income, $[a]	66,057 (10,998)	67,239 * (11,823)	65,247 (9,744)	65,618 (10,914)	65,671 (11,321)	67,010 * (12,688)	64,807 (9,833)	65,148 (10,896)
Health insurance, %	85.3 (5.3)	85.5 (5.1) *	85.5 (4.8) *	85 (5.7)	90.9 (4.7)	90.9 (4.8)	91.2 (4.2) *	90.8 (5.0)
Unemployment rate, %	8.2 (3.4)	8.3 (3.4) *	8.8 (2.9) *	7.8 (3.6)	5.2 (2.4)	5.4 (2.5) *	5.4 (2.0) *	5.1 (2.5)
Mean resident age, y	40.8 (4.5)	40.6 (4.3) *	40.7 (3.9)	41.0 (4.8)	42.1 (4.9)	41.8 (4.9)	42.3 (4.4)	42.1 (5.2)
White residents, %	82.4 (17.4)	82.2 (16.8)	84.1 (16.1) *	81.6 (18.4)	80.8 (17.4)	80.3 (17.3)	82.7 (16.1) *	80.2 (18.0)
Market share of IP stays, %	35.7 (17.4)	35.9 (17.2)	34.6 (17.3) *	36.2 (17.5)	25.1 (15.9)	25.3 (15.9)	23.2 (15.6) *	25.8 (16.0)
Hospitals within 15 miles, No.	1.5 (0.9)	1.6 (1.2) *	1.5 (0.8) *	1.4 (0.7)	1.4 (0.8)	1.5 (0.9) *	1.5 (0.7) *	1.3 (0.7)
HHI score (scale of 0 to 10,000)	8,535 (2,178)	8,271 * (2,320)	8,477 * (2,197)	8,750 (2,041)	8,744 (2,043)	8,512 * (2,154)	8,647 * (2,121)	8,954 (1,901)

© 2022 Jiang HJ et al. *JAMA Health Forum.*

Characteristic	2007				2019			
	All rural hospitals in HCUP, 2007	Already affiliated in 2007	Became affiliated during the study period	Independent until closure or 2019	All rural hospitals in HCUP, 2019	Already affiliated in 2007	Became affiliated during the study period	Independent until 2019
**Utilization, No. (%)**								
Service line provision, %[b]								
Maternal/neonatal	1,164 (65.7)	412 (71.7) *	257 (66.4)	495 (61.1)	917 (55.1)	324 (59.9) *	193 (53.3)	400 (52.5)
MSUD	1,395 (78.7)	473 (82.3) *	322 (83.2) *	600 (74.1)	919 (55.2)	331 (61.2) *	217 (59.9) *	371 (48.7)
Injury	1,653 (93.3)	541 (94.1) *	373 (96.4) *	739 (91.2)	1,347 (80.9)	450 (83.2) *	299 (82.6)	598 (78.5)
Surgical	1,419 (80.1)	496 (86.3) *	326 (84.2) *	597 (73.7)	1,183 (71.1)	407 (75.2) *	267 (73.8) *	509 (66.8)
General medical	1,771 (99.9)	574 (99.8)	387 (100.0)	810 (100.0)	1,663 (99.9)	539 (99.6)	362 (100.0)	762 (100.0)

Abbreviations: HCUP, Healthcare Cost and Utilization Project; HHI, Herfindahl-Hirschman Index; IP, inpatient; MSUD, mental and/or substance use disorder; SD, standard deviation.

Note: Percentages may not add to 100% because of rounding or because of missing data.

[a] Income was adjusted for inflation. 2007 dollars are expressed as 2019 dollars.

[b] Defined as having five or more stays in the service line annually.

* *P* value < 0.05 from chi-square or t-test comparing affiliated with independent hospitals in 2007 or 2019.

Source: Agency for Healthcare Research and Quality, Healthcare Cost and Utilization Project, State Inpatient Databases, 2007-2019, and Hospital Market Structure Files, 2006 and 2018; American Community Survey, 2007-2019; American Hospital Association Annual Survey, 2007-2019; Centers for Medicare & Medicaid Services, Cost Reports, 2007-2019; Irving Levin Associates, Inc., Mergers and Acquisitions Database, 2007-2019; Kaiser Family Foundation; University of North Carolina Sheps Center, 2007-2019.

© 2022 Jiang HJ et al. *JAMA Health Forum.*

eFigure 1. Changes in Inpatient Utilization Before and After the Affiliation for Hospitals that Became Affiliated During the Study Period

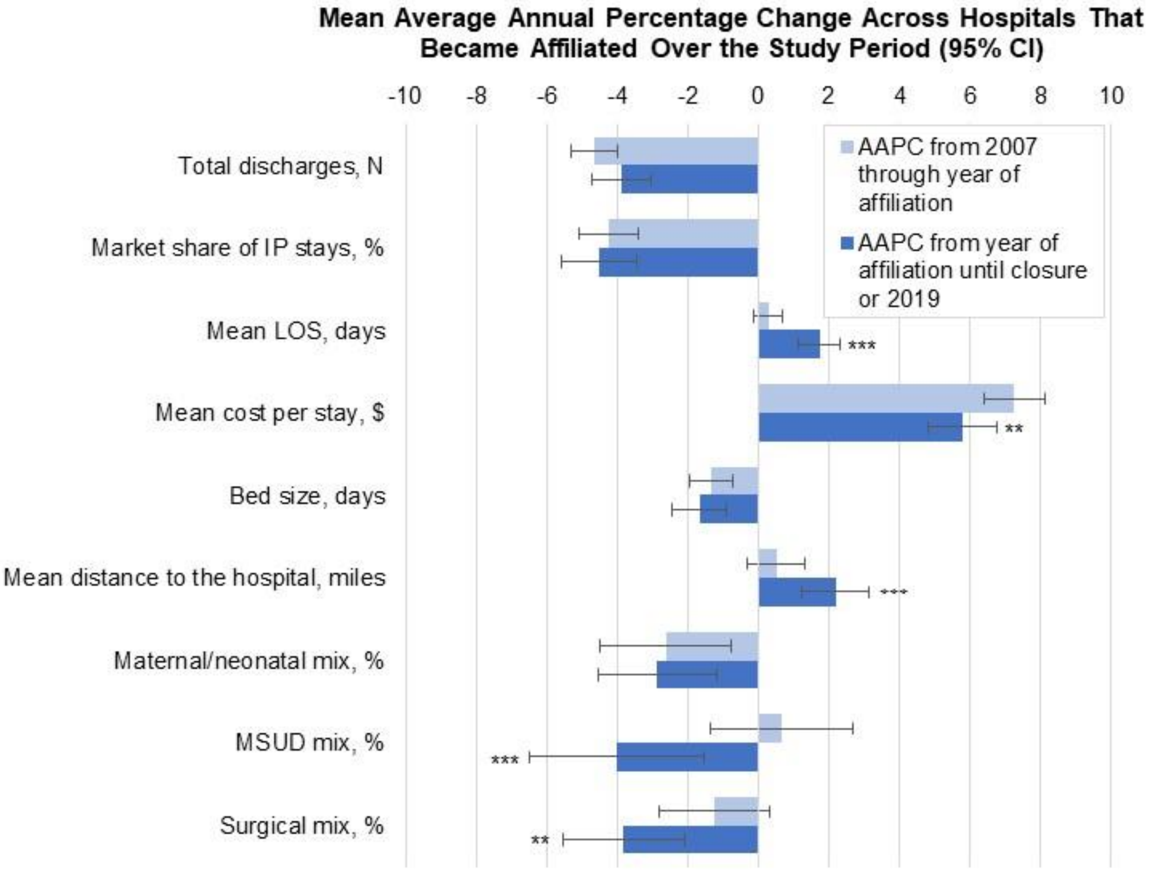

Abbreviations: CI, confidence interval; IP, inpatient; LOS, length of stay; MSUD, mental and/or substance use disorder.

Note: Excludes outlier hospitals where the average annual percentage change fell below the 1st percentile or above the 99th percentile. Average annual percentage change in utilization characteristics from 2007 until closure or 2019 was calculated for each hospital with nonmissing values in both years and a nonzero value in 2007.

* *P* < 0.10 for difference with independent hospitals

** *P* < 0.05 for difference with independent hospitals

*** *P* < 0.01 for difference with independent hospitals

Source: Agency for Healthcare Research and Quality, Healthcare Cost and Utilization Project, State Inpatient Databases, 2007-2019; American Hospital Association Annual Survey, 2007-2019; Centers for Medicare & Medicaid Services, Cost Reports, 2007-2019; Irving Levin Associates, Inc., Mergers and Acquisitions Database, 2007-2019.

© 2022 Jiang HJ et al. JAMA Health Forum.

eTable 4. Proportional Hazard Ratios Predicting Risk of Closure (Full Models)

Independent variable	Adjusted HR (95% CI)		
	All hospitals	Financially distressed in 2007	Financially stable in 2007
**Hospital characteristics**			
Affiliation	0.96 (0.60-1.52)	0.49 (0.26-0.92) **	2.36 (1.20-4.62) **
Financially distressed	1.98 (1.29-3.04) ***	—[a]	—[a]
Ownership			
Public	0.51 (0.30-0.88) **	0.21 (0.08-0.51) ***	0.96 (0.46-2.03)
Private nonprofit	[ref]	[ref]	[ref]
Private for-profit	1.55 (0.89-2.70)	0.76 (0.38-1.53)	4.08 (1.86-8.97) ***
Critical access hospital	0.12 (0.07-0.20)	0.15 (0.07-0.35)	0.06 (0.02-0.17)
Bed size, No.	1.00 (1.00-1.01) *	1.00 (1.00-1.01)	1.01 (1.00-1.02)
Hospital region			
Northeast	[ref]	[ref]	[ref]
Midwest	0.35 (0.14-0.88) **	0.20 (0.06-0.64) ***	0.67 (0.07-6.25)
South	0.34 (0.12-0.93) **	0.29 (0.07-1.25) *	0.45 (0.05-4.21)
West	0.53 (0.17-1.67)	0.54 (0.12-2.41)	0.40 (0.03-5.24)
In Medicaid expansion state	0.58 (0.33-1.02) *	0.46 (0.20-1.05) *	0.93 (0.41-2.08)
**Market characteristics**			
Mean household income, $[b]	1.00 (0.97-1.02)	1.00 (0.95-1.05)	0.98 (0.94-1.03)
Health insurance, %	1.01 (0.95-1.06)	0.99 (0.91-1.09)	1.01 (0.92-1.11)
Unemployment rate, %	1.03 (0.96-1.11)	1.01 (0.91-1.13)	1.04 (0.91-1.19)
Mean age, years	1.01 (0.94-1.08)	1.00 (0.90-1.11)	1.03 (0.93-1.14)
White residents, %	0.99 (0.98-1.01)	0.99 (0.97-1.01)	0.98 (0.95-1.01)
Market share of IP stays, %	0.98 (0.96-0.99) ***	0.98 (0.95-1.00) **	0.99 (0.96-1.02)
**Utilization characteristics**			
Total IP stays, No.[b]	0.75 (0.58-0.97) **	0.81 (0.58-1.14)	0.66 (0.45-0.98) **
Occupancy rate, %	0.23 (0.08-0.68) ***	0.16 (0.03-0.86) **	0.23 (0.04-1.25) *
Length of stay, mean days	1.02 (0.98-1.06)	1.02 (0.98-1.06)	0.98 (0.84-1.14)
Cost per stay, mean $[b]	1.00 (0.94-1.07)	1.05 (1.01-1.09) ***	1.00 (0.91-1.10)
Distance traveled, mean miles	0.98 (0.95-1.00) *	0.95 (0.91-1.00) **	0.99 (0.96-1.01)
Payer mix, %			
Medicare	0.98 (0.96-1.00) *	0.97 (0.94-0.99) **	0.99 (0.95-1.03)
Medicaid	—[c]	—[c]	—[c]
Private insurance	1.00 (0.97-1.03)	0.96 (0.92-1.00) **	1.03 (0.98-1.08)
Self-pay/no charge	0.98 (0.95-1.02)	0.93 (0.85-1.00) *	1.00 (0.95-1.05)
Other	0.98 (0.95-1.01)	0.96 (0.92-1.00) **	1.01 (0.96-1.05)
Service line mix, %			
Maternal/neonatal	0.95 (0.93-0.98) ***	0.94 (0.91-0.98) ***	0.96 (0.93-0.99) **
MSUD	0.97 (0.95-1.00) *	0.99 (0.95-1.03)	0.98 (0.94-1.03)

© 2022 Jiang HJ et al. JAMA Health Forum.

Independent variable	Adjusted HR (95% CI)		
	All hospitals	Financially distressed in 2007	Financially stable in 2007
Injury	0.93 (0.83-1.04)	0.83 (0.71-0.98) **	1.05 (0.94-1.16)
Surgical	0.94 (0.89-1.00) **	0.99 (0.92-1.07)	0.88 (0.81-0.95) ***
General medical	—[c]	—[c]	—[c]

Abbreviations: CI, confidence interval; HR, hazard ratio; IP, inpatient; MSUD, mental and/or substance use disorder.

[a] Not applicable in stratified model.

[b] Model parameters are expressed as the change in risk of closure per 1,000 unit increase in the independent variable; all other parameters are per 1 unit increase.

[c] Intentionally left out of the model because of multicollinearity (ie, the shares of each service line and each payer add to 100%).

* *P* < 0.10

** *P* < 0.05

*** *P* < 0.01

Source: Agency for Healthcare Research and Quality, Healthcare Cost and Utilization Project, State Inpatient Databases, 2007-2019, and Hospital Market Structure Files, 2006 and 2018; American Community Survey, 2007-2019; American Hospital Association Annual Survey, 2007-2019; Centers for Medicare & Medicaid Services, Cost Reports, 2007-2019; Irving Levin Associates, Inc., Mergers and Acquisitions Database, 2007-2019; Kaiser Family Foundation; University of North Carolina Sheps Center, 2007-2019.

© 2022 Jiang HJ et al. JAMA Health Forum.

eFigure 2. Unadjusted Survival Curves Measuring the Percentage of Hospitals Still Operating Through 2019, by Financial Distress in 2007

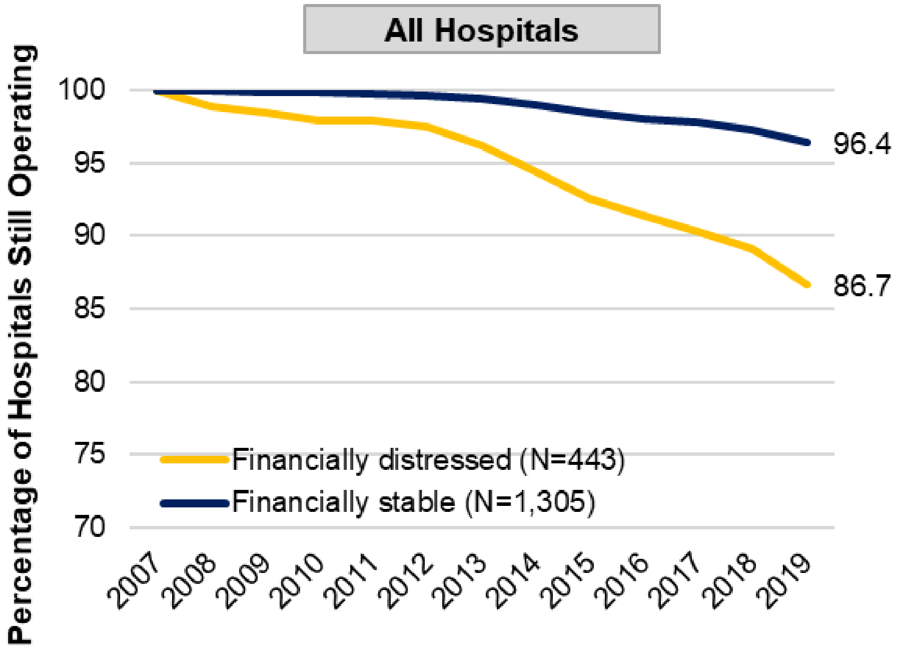

Source: Agency for Healthcare Research and Quality, Healthcare Cost and Utilization Project, State Inpatient Databases, 2007-2019; American Hospital Association Annual Survey, 2007-2019; Centers for Medicare & Medicaid Services, Cost Reports, 2007-2019; Irving Levin Associates, Inc., Mergers and Acquisitions Database, 2007-2019.

© 2022 Jiang HJ et al. JAMA Health Forum.

eFigure 3. Unadjusted Survival Curves Measuring the Percentage of Hospitals Still Operating Through 2019, by Ownership Overall and by Financial Distress in 2007

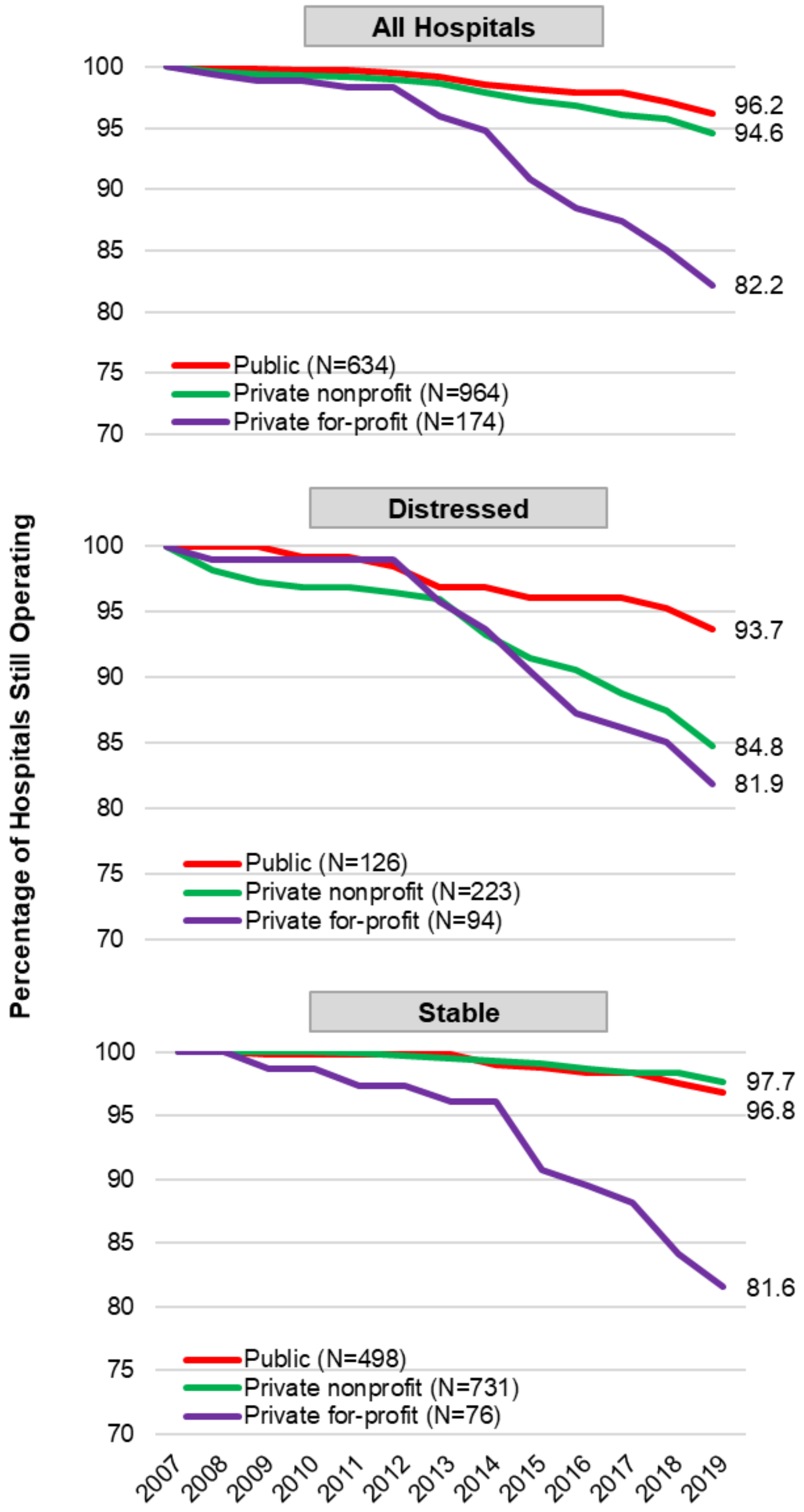

© 2022 Jiang HJ et al. JAMA Health Forum.

Source: Agency for Healthcare Research and Quality, Healthcare Cost and Utilization Project, State Inpatient Databases, 2007-2019; American Hospital Association Annual Survey, 2007-2019; Centers for Medicare & Medicaid Services, Cost Reports, 2007-2019; Irving Levin Associates, Inc., Mergers and Acquisitions Database, 2007-2019.

© 2022 Jiang HJ et al. JAMA Health Forum.

eFigure 4. Unadjusted Survival Curves Measuring the Percentage of Hospitals Still Operating Through 2019, by Critical Access Status Overall and by Financial Distress in 2007

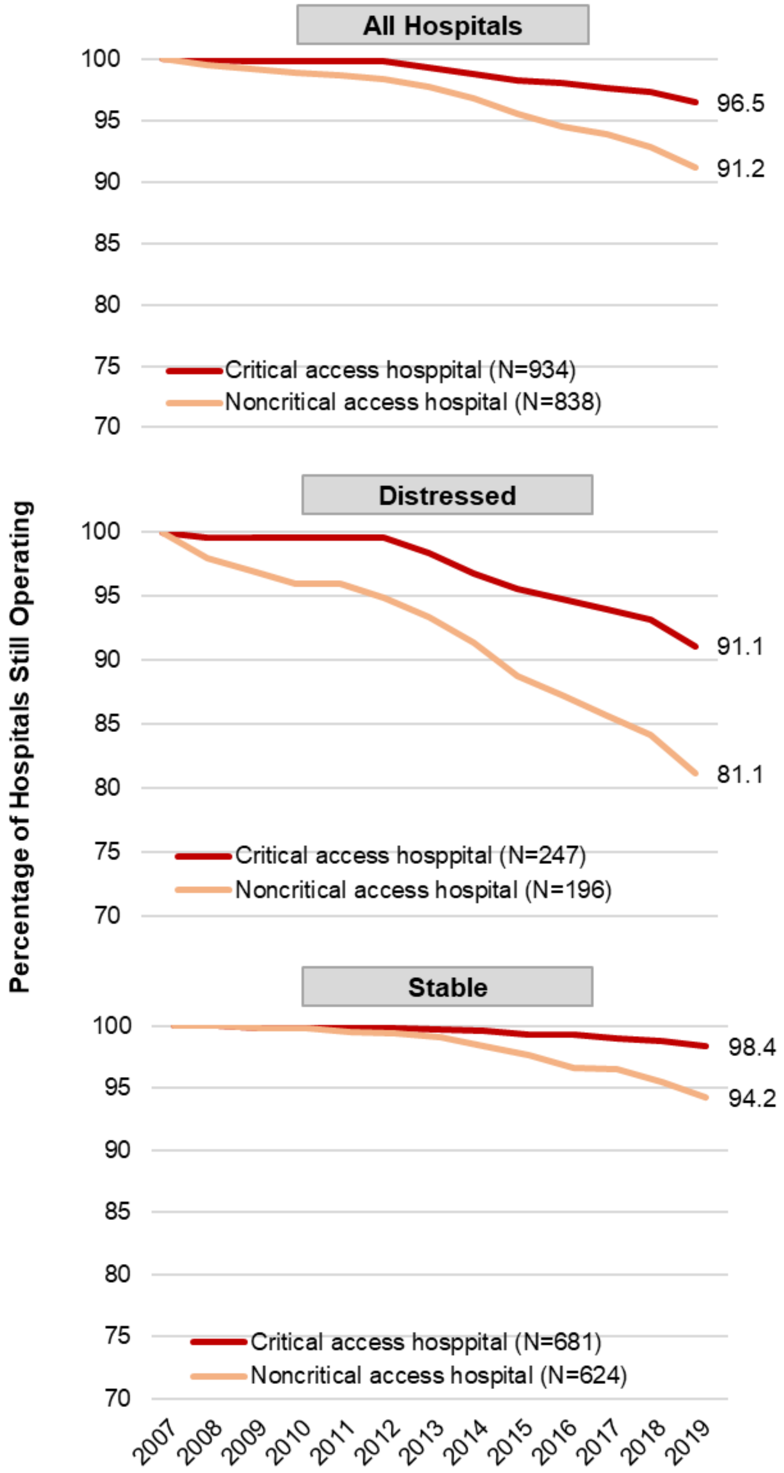

Source: Agency for Healthcare Research and Quality, Healthcare Cost and Utilization Project, State Inpatient Databases, 2007-2019; American Hospital Association Annual Survey, 2007-2019; Centers for Medicare & Medicaid Services, Cost Reports, 2007-2019; Irving Levin Associates, Inc., Mergers and Acquisitions Database, 2007-2019.

© 2022 Jiang HJ et al. JAMA Health Forum.

eFigure 5. Unadjusted Survival Curves Measuring the Percentage of Hospitals Still Operating Through 2019, by Region Overall and by Financial Distress in 2007

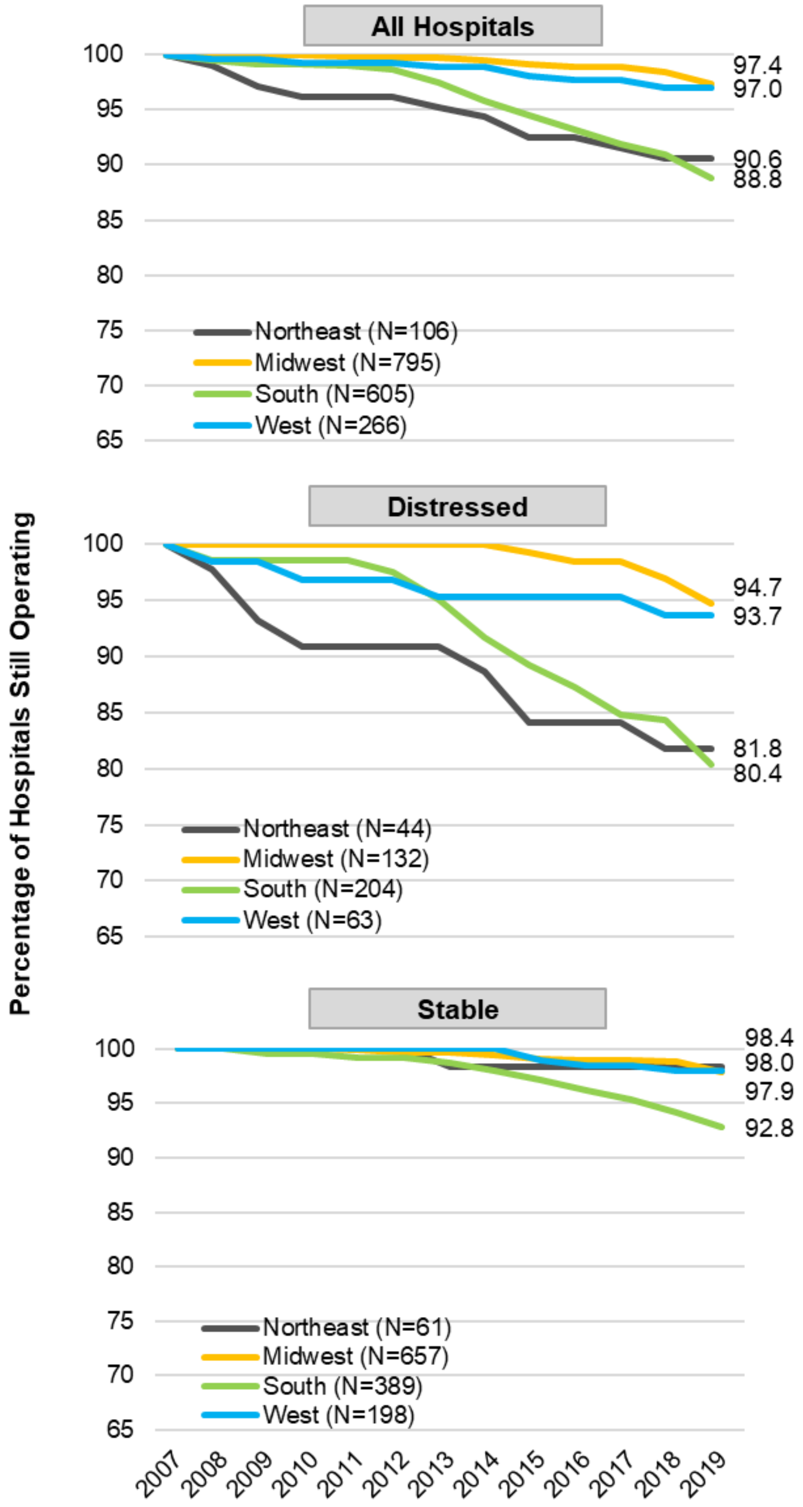

Source: Agency for Healthcare Research and Quality, Healthcare Cost and Utilization Project, State Inpatient Databases, 2007-2019; American Hospital Association Annual Survey, 2007-2019; Centers for Medicare & Medicaid Services, Cost Reports, 2007-2019; Irving Levin Associates, Inc., Mergers and Acquisitions Database, 2007-2019.

© 2022 Jiang HJ et al. JAMA Health Forum.
